# Entropy for q-rung linear diophantine fuzzy hypersoft set with its application in MADM

**DOI:** 10.1038/s41598-024-56252-6

**Published:** 2024-03-08

**Authors:** AN. Surya, J. Vimala, Nasreen Kausar, Željko Stević, Mohd Asif Shah

**Affiliations:** 1 Department of Mathematics, Alagappa University, Karaikudi, Tamilnadu India; 2 Department of Mathematics, Faculty of Arts and Science, Yildiz Technical University, Esenler, 34220 Istanbul Turkey; 3 School of Industrial Management Engineering, Korea University, 145 Anam-Ro, Seongbuk-Gu, Seoul, 02841 Korea; 4 Department of Economics, Kabridahar University, Kabridahar, 250 Somali Ethiopia; 5 Centre of Research Impact and Outcome, Chitkara University Institute of Engineering and Technology, Chitkara University, Rajpura, 140401 Punjab India; 6 Chitkara Centre for Research and Development, Chitkara University, Baddi, 174103 Himachal Pradesh India

**Keywords:** Mathematics and computing, Pure mathematics, Mathematics and computing, Pure mathematics

## Abstract

A notable advancement in fuzzy set theory is the q-rung linear diophantine fuzzy set. The soft set theory was expanded into the hypersoft set theory. By combining both the q-rung linear diophantine fuzzy set and hypersoft set, this study describes the notion of q-rung linear diophantine fuzzy hypersoft set that can handle multi sub-attributed q-rung linear diophantine fuzzy situations in the real world. Furthermore, some of its algebraic operations such as union, intersection and complement are described in this study. In addtion, the entropy measure of the q-rung linear diophantine fuzzy hypersoft set is established as it is helpful in determining the degree of fuzziness of q-rung linear diophantine fuzzy hypersoft sets. A multi-attribute decision making algorithm based on suggested entropy is presented in this study along with a numerical example of selecting a suitable wastewater treatment technology to demonstrate the effectiveness of the proposed algorithm in real-life situations. A comparative study was undertaken that describes the validity, robustness and superiority of the proposed algorithm and notions by discussing the advantages and drawbacks of existing theories and algorithms. Overall, this study describes a novel fuzzy extension that prevails over the existing ones and contributes to the real world with a valid real-life multi-attribute decision making algorithm that can cover many real-world problems that are unable to be addressed by the existing methodology.

## Introduction

To deal with the difficulties involved in multi-attribute decision making(MADM), the fuzzy set(FS) theory developed by Zadeh^1^ in 1965 is significant. It also offers a practical method for representing the fuzzy information. However, FS has a restricted capacity to represent a neutral state. Atanassov^2^ created the notion of the intuitionistic fuzzy set(IFS) to overcome these restrictions. The membership grade(MG) and non membership grade(NMG) are the two indices of the IFS, the IFSs MG and NMG totals should fall between [0,1]. Yager^3^ created the Pythagorean fuzzy set(PFS) as a result to ease issues, where MG^2^+NMG^2^ $\in$ [0,1]. In addition, Yager^4^ devised q-rung orthopair fuzzy sets(q-ROFS), where MG^q^+NMG^q^ $\in$ [0,1]. However, each of these concepts has disadvantages. Riaz and Hashmi^5^ devised the theory of the linear Diophantine fuzzy set(LDFS), which incorporates the idea of reference parameters(RPs) with the restriction that the sum of RPs should lie within the interval [0,1], to eliminate the disadvantages of the above mentioned concepts. Later, many researchers developed hybrids of LDFS such as linear diophantine fuzzy graphs^6^ and aggregation operators^7^ for LDFS. Even though LDFS is efficient in handling many real-life MADM problems its capability to handle real-life MADM problems is limited due to the restriction with RPs. To overcome the drawback of LDFS, the range of RPs was increased by qth powering the RPs and the concept of q-RLDFS was developed by Almagrabi^8^ as a specific extension of the IFS, q-ROFS, and LDFS with the restriction that the sum of qth power of RPs should lie within the interval [0,1]. However, all of these theories have certain limitations because of their lack of parametrization. Molodtsov^9^ created the concept of soft set(SS) theory, which addresses unpredictability in a parametric manner, to overcome the limits of parametrization. Maji et al.^10^ proposed the idea of the fuzzy soft set(FSS) by the integrating FS and SS. Similarly, SS theory was integrated with other extensions of FS theory such as IFS, PFS, q-ROFS and LDFS and obtained intuitionistic fuzzy soft set(IFSS), pythagorean fuzzy soft set(PFSS), q-rung orthopair fuzzy soft set(q-ROFSS) and linear diophantine fuzzy soft set(LDFSS) by Agman and Karatas^11^, Peng et al.^12^, Hussain et al.^13^ and Riaz et al.^14^ respectively. Subsequently, as a generalization of SS, Sanrandache^15^ created the concept of hypersoft set(HSS) by changing the function into a multi attributed function. Sanrandache^15^ also proposed the concepts of fuzzy hypersoft set(FHSS) and intuitionistic fuzzy hypersoft set(IFHSS) by combining HSS with FS and IFS. Later, Zulqarnain et al.^16^ proposed the pythagorean fuzzy hypersoft set(PFHSS) that combines PFS and HSS, likewise Khan et al.^17^ proposed the q-rung orthopair fuzzy hypersoft set(q-ROFHSS) that combines q-ROFS and HSS. Furthermore, many researchers have discussed various properties and decision making(DM) applications based on hybrid fuzzy and hybrid fuzzy hypersoft structures in different fields. For example, DM in areas such as myocardial infarction^18^, agri-drone^19^, hydogen production^20^, material selection^21,22^, supplier selection^23^, construction companies^24^ and thermal energy storage^25,26^.

In FS theory, entropy(ENT) is a key subject. The degree of fuzziness in the FSs is described by their ENT. Fuzzy ENT was initially presented by Zadeh^27^ in 1965. The fuzzy set ENT axiom construction was presented by Luca and Termini^28^, who also mentioned Shannon’s probability ENT and used it as a gauge of the amount of information. Kaufmann^29^ noted that one can determine the ENT of a fuzzy set by calculating the distance between the FS and the closest non-fuzzy set. Higashi and Klir^30^ used the distance from an FS to its complement. Trillas and Riera^31^ presented broad formulations of this ENT. Later, various ENT measures were extended to the different extensions of FS theory such as ENT measures for FSSs^32^, ENT measures for IFSs^33^, ENT measure for IFSSs^34^, ENT measures for PFSs^35^, ENT measures for PFSSs^36^, ENT measures for q-ROFSs^37^ and ENT measure for LDFS^38^. By calculating the fuzziness using ENTs, real-life DM problems can be handled better. This became a reason for many researchers to discuss various applications based on ENTs such as DM in Computer system security^39^, feature selection^40^ and DM in various other areas^41,42^.

There are several abbreviations in the paper. To ease readability, we have summarized the majority of the abbreviations in Table 1.

**Table 1:** List of abbreviation used in the study.

Abbreviation	Description
MADM	Multi-attributed decision making
FS	Fuzzy set
MG	Membership grade
NMG	Non membership grade
IFS	Intuitionistic fuzzy set
PFS	Pythagorean fuzzy set
q-ROFS	q-Rung orthopair fuzzy set
LDFS	Linear diophantine fuzzy set
RPs	Reference parameters
q-RLDFS	q-Rung linear diophantine fuzzy set
SS	Soft set
FSS	Fuzzy soft set
IFSS	Intuitionistic fuzzy soft set
PFSS	Pythagorean fuzzy soft set
q-ROFSS	q-Rung orthopair fuzzy soft set
LDFSS	Linear diophantine fuzzy soft set
HSS	Hypersoft set
FHSS	Fuzzy hypersoft set
IFHSS	Intuitionistic fuzzy hypersoft set
PFHSS	Pythagorean fuzzy hypersoft set
q-ROFHSS	q-Rung orthopair fuzzy hypersoft set
q-RLDFHSS	q-Rung linear diophantine fuzzy hypersoft set
WWTT	Waste water treatment technology
WWT	Waste water treatment
DM	Decision making
ENT	Entropy
MBR	Membrane bioreactor
SBR	Sequential batch reactor
FAB	Fluidized aerobic bed reactor

The research gaps are as follows:

- From the literature review, we can observe that even though there are many parametric DM studies under different fuzzy structures, when it comes to q-RLDFS with parametric information, it is difficult to demonstrate with the existing literature,which led to a theoretical research gap that is lacking of theory that can handle q-RLDFS with parametric information.
- Although there are various DM approaches and methods, ENT plays an important role in measuring fuzziness in fuzzy sets. Further from the literature review, we can notice that it is arduous to measure fuzziness in q-RLDFS with parametric information from the existing literature,which led to a research gap by lacking of theory that can measure fuzziness of q-RLDFS with parametric information.

The motivations of the study are as follows:

- The motivation is to fill the theoretical research gap by establishing theories that have the ability to handle parametric situations even in a q-RLDF environment and to measure the fuzziness in those situations.
- Because multiple sub-attributes must be dealt with simultaneously in many real-world MADM problems in the q-RLDFS environment, which are challenging to address with the current theories, this motivates the study to propose a MADM approach that has the ability to handle situations even in such challenging environments.

The main objectives of this work are listed below:

- To introduce the notion of q-rung linear diophantine fuzzy hypersoft set (q-RLDFHSS), which has great potential in handling multiple sub-attributed situations in q-RLDF environments.
- To introduce the ENT of q-RLDFHSS, which has the ability to measure the fuzziness of q-RLDFHSS.
- To propose a valid MADM approach based on ENT of q-RLDFHSS.
- To give a suitable numerical example for the proposed MADM approach.

The core contribution of the work are as follows:

- By fusing both q-RLDFS and HSS a new notion called q-RLDFHSS is presented in this study along with some of its algebraic operations such as union, intersection, complement and their properties.
- The ENT of q-RLDFHSS, which has the potential to measure the fuzziness of q-RLDFHSS, is also presented in this study.
- A MADM algorithm is imparted in this study based on the proposed ENT which has the potential in handling real-life MADM problems that are unable by the existing theories in the literature review.
- To demonstrate the effectiveness of the proposed MADM algorithm, a real-world situation of selecting a suitable wastewater treatment technology(WWTT) by considering multiple sub-parameters under q-RLDFHS environment is illustrated as a numerical example for the proposed MADM approach, since wastewater treatment(WWT) is a process that cleans up and removes impurities in wastewater so that it can be transformed into effluent and sent back into the water cycle. Effluent has a minimal negative influence on the environment after entering the water cycle or can be recycled for various uses. Furthermore, an elaborated general outlook on WWTT is provided in the numerical example section.
- To demonstrate the validity, robustness and superiority of the proposed notions and MADM approach a comparative study is presented. Additionally, minor limitations of the proposed study are discussed.

**Figure 1:**
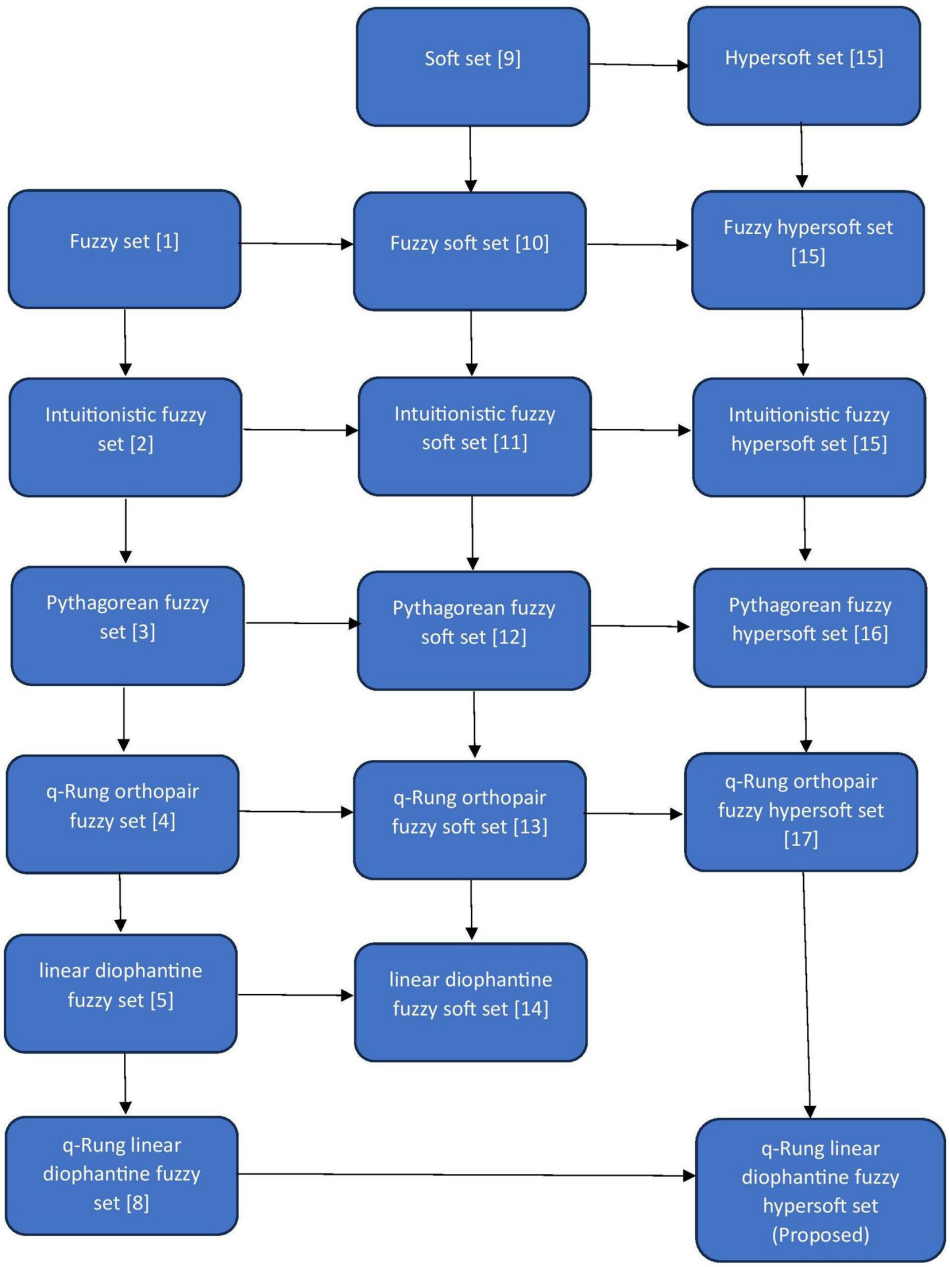
Fuzzy set extensions.

The novelty of the proposed study is as follows:

- The proposed q-RLDFHSS is an novel extension formed by combining both q-RLDFS and HSS which has more potential in handling real-life problems than the existing fuzzy extensions. Further, to understand the novelty of the proposed q-RLDFHSS easily, a map of existing fuzzy extensions along with the proposed extension is shown in Fig. 1.

The organization of the paper is as follows:

The necessary introductory definitions and notations are presented in “Preliminaries” section. Section “q-Rung linear diophantine fuzzy hypersoft set” contains the definitions of the proposed q-RLDFHSS, along with some of its fundamental operations and its ENT. From the proposed theories in “Section 3”, a MADM algorithm based on the suggested ENT is presented in “Section 4” to effectively address MADM issues. The MADM problem of selecting the WWTT was used to demonstrate the effectiveness of the proposed algorithm. Then, to show the validation of the MADM approach proposed in “Application”’ section and the robustness and superiority of the proposed method a comprehensive comparative analysis has been undertaken in “Comparative study” section. The article’s conclusion and a discussion of future works are provided in ”Conclusion and future studies” section.

## Preliminaries

In this section the notations and definitions helpful for the paper are provided.

### Definition 0.1

^1^ Let $\Re$ be a universal set. A FS ${\mathcal {F}}$ is defined as

$$\begin{aligned} {\mathcal {F}}=\{({\mathfrak {r}}, \Delta _{{\mathcal {F}}}({\mathfrak {r}}))| {\mathfrak {r}}\in \Re \} \end{aligned}$$

where, $\Delta _{{\mathcal {F}}}({\mathfrak {r}})\in$ [0,1] is the MG of ${\mathfrak {r}}\in \Re$.

### Definition 0.2

^2^ Let $\Re$ be a universal set. A IFS ${\mathcal {I}}$ is defined as

$$\begin{aligned} {\mathcal {I}}=\{({\mathfrak {r}}, \Delta _{{\mathcal {I}}}({\mathfrak {r}}), \nabla _{{\mathcal {I}}}({\mathfrak {r}}))| {\mathfrak {r}}\in \Re \} \end{aligned}$$

where, $\Delta _{{\mathcal {I}}}({\mathfrak {r}})$ and $\nabla _{{\mathcal {I}}}({\mathfrak {r}})\in$ [0,1] are MG and NMG satisfying 0$\le \Delta _{{\mathcal {I}}}({\mathfrak {r}})+\nabla _{{\mathcal {I}}}({\mathfrak {r}}) \le$ 1.

### Definition 0.3

^8^ Let $\Re$ be a universal set. A q-RLDFS ${\mathcal {Q}}$ is defined as

$$\begin{aligned} {\mathcal {Q}}=\{({\mathfrak {r}}, \langle \Delta _{{\mathcal {Q}}}({\mathfrak {r}}), \nabla _{{\mathcal {Q}}}({\mathfrak {r}})\rangle , \langle \rtimes _{{\mathcal {Q}}}({\mathfrak {r}}), \ltimes _{{\mathcal {Q}}}({\mathfrak {r}})\rangle | {\mathfrak {r}}\in \Re \} \end{aligned}$$

where, $\Delta _{{\mathcal {Q}}}({\mathfrak {r}}), \nabla _{{\mathcal {Q}}}({\mathfrak {r}}), \rtimes _{{\mathcal {Q}}}({\mathfrak {r}})$ and $\ltimes _{{\mathcal {Q}}}({\mathfrak {r}})\in$ [0,1] are MG, NMG and their corresponding RPs,

respectively satisfying 0$\le \rtimes _{{\mathcal {Q}}}^{q}({\mathfrak {r}})+\ltimes _{{\mathcal {Q}}}^{q}({\mathfrak {r}})\le$ 1 and 0$\le \rtimes _{{\mathcal {Q}}}^{q}({\mathfrak {r}})\Delta _{{\mathcal {Q}}}({\mathfrak {r}})+\ltimes _{{\mathcal {Q}}}^{q}({\mathfrak {r}})\nabla _{{\mathcal {Q}}}({\mathfrak {r}}) \le$ 1 $\forall {\mathfrak {r}}\in \Re$, q$\ge$ 1.

### Definition 0.4

^9^ Let $\Re$ be a universal set, ${\mathfrak {E}}$ be set of attributes and ${\mathfrak {A}}\subseteq {\mathfrak {E}}$. Then SS is a pair$(\Xi ,{\mathfrak {A}})$ represented by the mapping

$$\begin{aligned} \Xi : {\mathfrak {A}} \rightarrow P(\Re ) \end{aligned}$$

Where, P$(\Re )$ is set of all subset of $\Re$.

### Definition 0.5

^10^ Let $\Re$ be a universal set, ${\mathfrak {E}}$ be set of attributes and ${\mathfrak {A}}\subseteq {\mathfrak {E}}$. Then FSS is a pair$(\Xi ,{\mathfrak {A}})$ represented by the mapping

$$\begin{aligned} \Xi : {\mathfrak {A}} \rightarrow FP(\Re ) \end{aligned}$$

Where, FP$(\Re )$ is set of all fuzzy subset of $\Re$.

### Definition 0.6

^15^ Let $\Re$ be a universal set, ${\mathfrak {E}}_{1}, {\mathfrak {E}}_{2},..., {\mathfrak {E}}_{n}$ be the corresponding attribute values of n different attributes ${\mathfrak {e}}_{1}, {\mathfrak {e}}_{2},..., {\mathfrak {e}}_{n}$ respectively such that ${\mathfrak {E}}_{\imath }\cap {\mathfrak {E}}_{\jmath }= \emptyset$ for $\imath \ne \jmath$ and ${\mathfrak {A}}_{\imath } \subseteq {\mathfrak {E}}_{\imath }$ for $\imath$=1,2,...,n and $\mho _{1}= {\mathfrak {A}}_{1} \times {\mathfrak {A}}_{2} \times ... \times {\mathfrak {A}}_{n} \subseteq {\mathfrak {E}}_{1}\times {\mathfrak {E}}_{2}\times ... \times {\mathfrak {E}}_{n}$. Then HSS is a pair$(\Xi ,\mho _{1})$ represented by the mapping

$$\begin{aligned} \Xi : \mho _{1} \rightarrow P(\Re ) \end{aligned}$$

It can be written as

$$(\Xi ,\mho _{1})=\{(\omega , \Xi (\omega )): \omega \in \mho _{1}, \Xi (\omega ) \in P(\Re )\}$$.

### Definition 0.7

^15^ Let $\Re$ be a universal set, ${\mathfrak {E}}_{1}, {\mathfrak {E}}_{2},..., {\mathfrak {E}}_{n}$ be the corresponding attribute values of n different attributes ${\mathfrak {e}}_{1}, {\mathfrak {e}}_{2},..., {\mathfrak {e}}_{n}$ respectively such that ${\mathfrak {E}}_{\imath }\cap {\mathfrak {E}}_{\jmath }= \emptyset$ for $\imath \ne \jmath$ and ${\mathfrak {A}}_{\imath } \subseteq {\mathfrak {E}}_{\imath }$ for $\imath$=1,2,...,n and $\mho _{1}= {\mathfrak {A}}_{1} \times {\mathfrak {A}}_{2} \times ... \times {\mathfrak {A}}_{n} \subseteq {\mathfrak {E}}_{1}\times {\mathfrak {E}}_{2}\times ... \times {\mathfrak {E}}_{n}$. Then IFHSS is a pair$(\Xi ,\mho _{1})$ represented by the mapping

$$\begin{aligned} \Xi : \mho _{1} \rightarrow IFP(\Re ) \end{aligned}$$

Where, IFP$(\Re )$ is the set of all IF subset of $\Re$

It can be written as

$$(\Xi ,\mho _{1})=\{(\omega , \Xi (\omega )): \omega \in \mho _{1}, \Xi (\omega ) \in IFP(\Re )\}$$.

### Definition 0.8

^32^ A real valued map ${\mathscr {E}}: (\Xi ,{\mathfrak {A}}) \rightarrow [0,+\infty )$ is said to be ENT on FSS, if ${\mathscr {E}}$ satisfies the conditions

(i) ${\mathscr {E}}(\Xi ,{\mathfrak {A}})$=0 if $(\Xi ,{\mathfrak {A}})$ is SS
(ii) ${\mathscr {E}}(\Xi ,{\mathfrak {A}})$=1 if $\Xi ({\mathfrak {a}})$=[0.5] for any ${\mathfrak {a}}\in {\mathfrak {A}}$, where [0.5] is the FS having MG [0.5]$({\mathfrak {r}})= 0.5 \forall {\mathfrak {r}}\in \Re$
(iii) If $(\Xi _{1},{\mathfrak {A}})$ is crisp set than that of $(\Xi _{2},{\mathfrak {A}})$ which is, for ${\mathfrak {a}}\in {\mathfrak {A}}$ and ${\mathfrak {r}}\in \Re$, $\Xi _{1}({\mathfrak {a}})({\mathfrak {r}})\le \Xi _{2}({\mathfrak {a}})({\mathfrak {r}})$ if $\Xi _{2}({\mathfrak {a}})({\mathfrak {r}})\le$ 0.5 and $\Xi _{2}({\mathfrak {a}})({\mathfrak {r}})\le \Xi _{1}({\mathfrak {a}})({\mathfrak {r}})$ if $\Xi _{2}({\mathfrak {a}})({\mathfrak {r}})\ge$ 0.5. Then ${\mathscr {E}}(\Xi _{1},{\mathfrak {A}})\le {\mathscr {E}}(\Xi _{2},{\mathfrak {A}})$
(iv) ${\mathscr {E}}(\Xi ,{\mathfrak {A}})$= ${\mathscr {E}}(\Xi ^{c},{\mathfrak {A}})$, where $(\Xi ^{c},{\mathfrak {A}})$ is the complement of FSS $(\Xi ,{\mathfrak {A}})$, given by $\Xi ^{c}({\mathfrak {a}})= (\Xi ({\mathfrak {a}}))^{c}$ for each ${\mathfrak {a}}\in {\mathfrak {A}}$.

## q-Rung linear diophantine fuzzy hypersoft set

In this section, the notion of q-RLDFHSS is established using some of its basic algebraic operations and the ENT for q-RLDFHSS is described.

### Definition 0.9

Let $\Re$ be a universal set, ${\mathfrak {E}}_{1}, {\mathfrak {E}}_{2},..., {\mathfrak {E}}_{n}$ be the corresponding attribute values of n different attributes ${\mathfrak {e}}_{1}, {\mathfrak {e}}_{2},..., {\mathfrak {e}}_{n}$ respectively such that ${\mathfrak {E}}_{\imath }\cap {\mathfrak {E}}_{\jmath }= \emptyset$ for $\imath \ne \jmath$ and ${\mathfrak {A}}_{\imath } \subseteq {\mathfrak {E}}_{\imath }$ for $\imath$=1,2,...,n and $\mho _{1}= {\mathfrak {A}}_{1} \times {\mathfrak {A}}_{2} \times ... \times {\mathfrak {A}}_{n} \subseteq {\mathfrak {E}}_{1}\times {\mathfrak {E}}_{2}\times ... \times {\mathfrak {E}}_{n}$. Then q-Rung linear diophantine fuzzy hypersoft set over $\Re$(q-RLDFHSS($\Re$)) is a pair$(\Xi ,\mho _{1})$ represented by the mapping

$$\begin{aligned} \Xi : \mho _{1} \rightarrow q-RLDFP(\Re ) \end{aligned}$$

It can be written as $(\Xi ,\mho _{1})=\{(\omega , \Xi (\omega )): \omega \in \mho _{1}, \Xi (\omega ) \in q-RLDFP(\Re )\}$

Where, q-RLDFP$(\Re )$ is the set of all q-RLDF subset of $\Re$ and q-RLDFHS Number(q-RLDFHSN)

$\Xi _{{\mathfrak {r}}_{p}}(\omega _{s})$= $\{\langle \Delta _{\Xi (\omega _{s})}({\mathfrak {r}}_{p}),\nabla _{\Xi (\omega _{s})}({\mathfrak {r}}_{p}) \rangle , \langle \rtimes _{\Xi (\omega _{s})}({\mathfrak {r}}_{p}),\ltimes _{\Xi (\omega _{s})}({\mathfrak {r}}_{p}) \rangle | {\mathfrak {r}}_{p} \in \Re \;and\;\omega _{s} \in \mho _{1} \}$ can be express as

$$\mathbb {j}_{\omega _{ps}}$$=$$\{\langle \Delta _{\omega _{ps}},\nabla _{\omega _{ps}} \rangle , \langle \rtimes _{\omega _{ps}},\ltimes _{\omega _{ps}} \rangle \}$$.

### Example 1

Let $\Re =\{{\mathfrak {r}}_{1},{\mathfrak {r}}_{2},{\mathfrak {r}}_{3}\}$ be the set of boats also consider the attributes ${\mathfrak {e}}_{1}$= cost, ${\mathfrak {e}}_{2}$= engine, ${\mathfrak {e}}_{3}$= hull and ${\mathfrak {E}}_{1}=\{$ Purchasing cost$({\mathfrak {e}}_{11})$, Maintenance cost$({\mathfrak {e}}_{12})\}$, ${\mathfrak {E}}_{2}=\{$ inboard engine$({\mathfrak {e}}_{21})$, outboard engine$({\mathfrak {e}}_{22})\}$, ${\mathfrak {E}}_{3}=\{$ planing hull$({\mathfrak {e}}_{31})\}$ be their corresponding attribute values,

Suppose ${\mathfrak {A}}_{\imath } \subseteq {\mathfrak {E}}_{\imath }$ for each $\imath$ = 1,2,3

Let ${\mathfrak {A}}_{\imath }$=${\mathfrak {E}}_{\imath }$ for each $\imath$ = 1,2,3. Then

$\mho _{1}$= ${\mathfrak {A}}_{1} \times {\mathfrak {A}}_{2} \times {\mathfrak {A}}_{3}$ =$\{\omega _{1}=({\mathfrak {e}}_{11},{\mathfrak {e}}_{21},{\mathfrak {e}}_{31}), \omega _{2}=({\mathfrak {e}}_{11},{\mathfrak {e}}_{22},{\mathfrak {e}}_{31}), \omega _{3}=({\mathfrak {e}}_{12},{\mathfrak {e}}_{21},{\mathfrak {e}}_{31}), \omega _{4}=({\mathfrak {e}}_{12},{\mathfrak {e}}_{22},{\mathfrak {e}}_{31})\}$ The classification of attributes is given as follows

- The attribute “Cost” and its attribute values illustrates that the alternative is cheap or not cheap.
- The attribute “Engine” and its attribute values illustrates that the alternative is high power or low power.
- The attribute “Hull” and its attribute values illustrates that the alternative is convenient or not convenient.

Then the cartesian product of sub-attributes illustrates that the alternative is (cheap, high power, convenient) all together or (not cheap, low power, not convenient) all together

The characteristic of this q-RLDFHSS is $(\langle$ MG, NMG $\rangle , \langle$ (cheap, high power, convenient), (not cheap, low power, not convenient)$\rangle )$ $\forall \omega _{s}\in \mho _{1}$.

Then, q-RLDFHSS ($\Xi ,\mho _{1}$) may be expressed as

$$\begin{aligned} (\Xi ,\mho _{1})=&\Biggl \{\biggl \langle \omega _{1},\left( \frac{{\mathfrak {r}}_{1}}{\langle (0.7,0.6),(0.6,0.6)\rangle }, \frac{{\mathfrak {r}}_{2}}{\langle (0.6,0.6),(0.5,0.4)\rangle }, \frac{{\mathfrak {r}}_{3}}{\langle (0.7,0.5),(0.6,0.5)\rangle }\right) \biggl \rangle ,\\&\;\;\biggl \langle \omega _{2},\left( \frac{{\mathfrak {r}}_{1}}{\langle (0.8,0.7),(0.7,0.4)\rangle }, \frac{{\mathfrak {r}}_{2}}{\langle (0.5,0.4),(0.6,0.7)\rangle }, \frac{{\mathfrak {r}}_{3}}{\langle (0.7,0.5),(0.5,0.7)\rangle }\right) \biggl \rangle ,\\&\;\;\biggl \langle \omega _{3},\left( \frac{{\mathfrak {r}}_{1}}{\langle (0.6,0.5),(0.5,0.4)\rangle }, \frac{{\mathfrak {r}}_{2}}{\langle (0.8,0.7),(0.6,0.6)\rangle }, \frac{{\mathfrak {r}}_{3}}{\langle (0.5,0.4),(0.5,0.4)\rangle }\right) \biggl \rangle ,\\&\;\;\biggl \langle \omega _{4},\left( \frac{{\mathfrak {r}}_{1}}{\langle (0.5,0.6),(0.4,0.5)\rangle }, \frac{{\mathfrak {r}}_{2}}{\langle (0.6,0.6),(0.7,0.5)\rangle }, \frac{{\mathfrak {r}}_{3}}{\langle (0.8,0.6),(0.6,0.4)\rangle }\right) \biggl \rangle \Biggl \} \end{aligned}$$

We will assume that q=3

The tabular form of q-RLDFHSS is shown in Table 2

**Table 2:** The tabular form of q-RLDFHSS ($\Xi ,\mho _{1}$).

($\Xi ,\mho _{1}$)	${\mathfrak {r}}_{1}$	${\mathfrak {r}}_{2}$	${\mathfrak {r}}_{3}$
$\omega _{1}$	$\langle$(0.7,0.6),(0.6,0.6)$\rangle$	$\langle$(0.6,0.6),(0.5,0.4)$\rangle$	$\langle$(0.7,0.5),(0.6,0.5)$\rangle$
$\omega _{2}$	$\langle$(0.8,0.7),(0.7,0.4)$\rangle$	$\langle$(0.5,0.4),(0.6,0.7)$\rangle$	$\langle$(0.7,0.5),(0.5,0.7)$\rangle$
$\omega _{3}$	$\langle$(0.6,0.5),(0.5,0.4)$\rangle$	$\langle$(0.8,0.7),(0.6,0.6)$\rangle$	$\langle$(0.5,0.4),(0.5,0.4)$\rangle$
$\omega _{4}$	$\langle$(0.5,0.6),(0.4,0.5)$\rangle$	$\langle$(0.6,0.6),(0.7,0.5)$\rangle$	$\langle$(0.8,0.6),(0.6,0.4)$\rangle$

### Definition 0.10

Let $(\Xi _{1},\mho _{1}),(\Xi _{2},\mho _{2})\in$ q-RLDFHSS($\Re$), then $(\Xi _{1},\mho _{1})$ is said to be q-RLDFHS subset of $(\Xi _{2},\mho _{2})$, if

(i) $$\mho _{1}\subseteq \mho _{2}$$

(ii) $$\forall \omega \in \mho _{1},\;\Xi _{1}(\omega )\subseteq \Xi _{2}(\omega )$$

i.e) $$\Delta _{\Xi _{1}(\omega )}({\mathfrak {r}}_{p}) \le \Delta _{\Xi _{2}(\omega )}({\mathfrak {r}}_{p}), \nabla _{\Xi _{2}(\omega )}({\mathfrak {r}}_{p}) \le \nabla _{\Xi _{1}(\omega )}({\mathfrak {r}}_{p}), \rtimes _{\Xi _{1}(\omega )}({\mathfrak {r}}_{p}) \le \rtimes _{\Xi _{2}(\omega )}({\mathfrak {r}}_{p})$$ and

$$\ltimes _{\Xi _{2}(\omega )}({\mathfrak {r}}_{p}) \le \ltimes _{\Xi _{1}(\omega )}({\mathfrak {r}}_{p}) \forall {\mathfrak {r}}_{p}\in \Re $$.

### Definition 0.11

A q-RLDFHSS $(\Xi ,\mho _{1})$ over $\Re$ is said to be null q-RLDFHSS if

$\Delta _{\Xi (\omega _{s})}({\mathfrak {r}}_{p})$=$\rtimes _{\Xi (\omega _{s})}({\mathfrak {r}}_{p})$=0 and $\nabla _{\Xi (\omega _{s})}({\mathfrak {r}}_{p})$=$\ltimes _{\Xi (\omega _{s})}({\mathfrak {r}}_{p})$=1 $\forall \omega _{s}\in \mho _{1}$ and ${\mathfrak {r}}_{p}\in \Re$ and it is denoted by $(\Xi ,\mho _{1})_{\emptyset }$.

### Definition 0.12

A q-RLDFHSS $(\Xi ,\mho _{1})$ over $\Re$ is said to be absolute q-RLDFHSS if

$\Delta _{\Xi (\omega _{s})}({\mathfrak {r}}_{p})$=$\rtimes _{\Xi (\omega _{s})}({\mathfrak {r}}_{p})$=1 and $\nabla _{\Xi (\omega _{s})}({\mathfrak {r}}_{p})$=$\ltimes _{\Xi (\omega _{s})}({\mathfrak {r}}_{p})$=0 $\forall \omega _{s}\in \mho _{1}$ and ${\mathfrak {r}}_{p}\in \Re$ and it is denoted by $(\Xi ,\mho _{1})_{U}$.

### Definition 0.13

Let

$(\Xi ,\mho _{1})=\biggl \{ \biggl (\omega , \biggl \langle \langle \Delta _{\Xi (\omega )}({\mathfrak {r}}) ,\nabla _{\Xi (\omega )}({\mathfrak {r}}) \rangle ,\langle \rtimes _{\Xi (\omega )}({\mathfrak {r}}), \ltimes _{\Xi (\omega )}({\mathfrak {r}}) \rangle \biggl \rangle \biggl ) | {\mathfrak {r}} \in \Re \;and\;\omega \in \mho _{1} \biggl \}$ be a q-RLDFHSS($\Re$), then its complement is defined and denoted as follows

$$(\Xi ,\mho _{1})^{c}=\biggl \{ \biggl (\omega , \biggl \langle \langle \nabla _{\Xi (\omega )}({\mathfrak {r}}) ,\Delta _{\Xi (\omega )}({\mathfrak {r}}) \rangle ,\langle \ltimes _{\Xi (\omega )}({\mathfrak {r}}), \rtimes _{\Xi (\omega )}({\mathfrak {r}}) \rangle \biggl \rangle \biggl ) | {\mathfrak {r}} \in \Re \;and\;\omega \in \mho _{1} \biggl \}$$.

### Theorem 0.14

Let $(\Xi ,\mho _{1})\in$ q-RLDFHSS($\Re$), then

(i) $$((\Xi ,\mho _{1})^{c})^{c}$$=$$(\Xi ,\mho _{1})$$

(ii) $$((\Xi ,\mho _{1})_{\emptyset })^{c}$$=$$(\Xi ,\mho _{1})_{U}$$

(iii) $$((\Xi ,\mho _{1})_{U})^{c}$$=$$(\Xi ,\mho _{1})_{\emptyset }$$.

### Proof

Proof is obvious $\square$

### Definition 0.15

Let $(\Xi _{1},\mho _{1}),(\Xi _{2},\mho _{2})\in$ q-RLDFHSS($\Re$), then the intersection of $(\Xi _{1},\mho _{1})$ and $(\Xi _{2},\mho _{2})$ is defined as $(\Xi _{1},\mho _{1})\cap (\Xi _{2},\mho _{2})$= $(\Xi _{3},\mho _{3})$, where $\mho _{3}=\mho _{1}\cap \mho _{2}$ and

$$\begin{aligned} (\Xi _{3},\mho _{3})=\biggl \{ \biggl (&\omega , \biggl \langle \langle Min(\Delta _{\Xi _{1}(\omega )}({\mathfrak {r}}),\Delta _{\Xi _{2}(\omega )}({\mathfrak {r}})) ,Max(\nabla _{\Xi _{1}(\omega )}({\mathfrak {r}}),\nabla _{\Xi _{2}(\omega )}({\mathfrak {r}})) \rangle ,\\&\;\;\;\;\;\;\;\;\;\langle Min(\rtimes _{\Xi _{1}(\omega )}({\mathfrak {r}}),\rtimes _{\Xi _{2}(\omega )}({\mathfrak {r}})) ,Max(\ltimes _{\Xi _{1}(\omega )}({\mathfrak {r}}),\ltimes _{\Xi _{2}(\omega )}({\mathfrak {r}})) \rangle \biggl \rangle \biggl ) | {\mathfrak {r}} \in \Re \;and\;\omega \in \mho _{3} \biggl \} \end{aligned}$$

### Definition 0.16

Let $(\Xi _{1},\mho _{1}),(\Xi _{2},\mho _{2})\in$ q-RLDFHSS($\Re$), then the union of $(\Xi _{1},\mho _{1})$ and $(\Xi _{2},\mho _{2})$ is defined as $(\Xi _{1},\mho _{1})\cup (\Xi _{2},\mho _{2})$= $(\Xi _{3},\mho _{3})$, where $\mho _{3}=\mho _{1}\cup \mho _{2}$ and

$$\begin{aligned} \Delta _{\Xi _{3}(\omega )}({\mathfrak {r}})= {\left\{ \begin{array}{ll} \Delta _{\Xi _{1}(\omega )}({\mathfrak {r}}) &{} if \omega \in \mho _{1}-\mho _{2}\;and\;{\mathfrak {r}}\in \Re \\ \Delta _{\Xi _{2}(\omega )}({\mathfrak {r}}) &{} if \omega \in \mho _{2}-\mho _{1}\;and\;{\mathfrak {r}}\in \Re \\ Max\{ \Delta _{\Xi _{1}(\omega )}({\mathfrak {r}}),\Delta _{\Xi _{2}(\omega )}({\mathfrak {r}})\} &{} if \omega \in \mho _{1}\cap \mho _{2}\;and\;{\mathfrak {r}}\in \Re \\ \end{array}\right. } \\ \nabla _{\Xi _{3}(\omega )}({\mathfrak {r}})= {\left\{ \begin{array}{ll} \nabla _{\Xi _{1}(\omega )}({\mathfrak {r}}) &{} if \omega \in \mho _{1}-\mho _{2}\;and\;{\mathfrak {r}}\in \Re \\ \nabla _{\Xi _{2}(\omega )}({\mathfrak {r}}) &{} if \omega \in \mho _{2}-\mho _{1}\;and\;{\mathfrak {r}}\in \Re \\ Min\{ \nabla _{\Xi _{1}(\omega )}({\mathfrak {r}}),\nabla _{\Xi _{2}(\omega )}({\mathfrak {r}})\} &{} if \omega \in \mho _{1}\cap \mho _{2}\;and\;{\mathfrak {r}}\in \Re \\ \end{array}\right. } \\ \rtimes _{\Xi _{3}(\omega )}({\mathfrak {r}})= {\left\{ \begin{array}{ll} \rtimes _{\Xi _{1}(\omega )}({\mathfrak {r}}) &{} if \omega \in \mho _{1}-\mho _{2}\;and\;{\mathfrak {r}}\in \Re \\ \rtimes _{\Xi _{2}(\omega )}({\mathfrak {r}}) &{} if \omega \in \mho _{2}-\mho _{1}\;and\;{\mathfrak {r}}\in \Re \\ Max\{ \rtimes _{\Xi _{1}(\omega )}({\mathfrak {r}}),\rtimes _{\Xi _{2}(\omega )}({\mathfrak {r}})\} &{} if \omega \in \mho _{1}\cap \mho _{2}\;and\;{\mathfrak {r}}\in \Re \\ \end{array}\right. } \\ \ltimes _{\Xi _{3}(\omega )}({\mathfrak {r}})= {\left\{ \begin{array}{ll} \ltimes _{\Xi _{1}(\omega )}({\mathfrak {r}}) &{} if \omega \in \mho _{1}-\mho _{2}\;and\;{\mathfrak {r}}\in \Re \\ \ltimes _{\Xi _{2}(\omega )}({\mathfrak {r}}) &{} if \omega \in \mho _{2}-\mho _{1}\;and\;{\mathfrak {r}}\in \Re \\ Min\{ \ltimes _{\Xi _{1}(\omega )}({\mathfrak {r}}),\ltimes _{\Xi _{2}(\omega )}({\mathfrak {r}})\} &{} if \omega \in \mho _{1}\cap \mho _{2}\;and\;{\mathfrak {r}}\in \Re \\ \end{array}\right. } \end{aligned}$$

### Theorem 0.17

Let $(\Xi _{1},\mho _{1}),(\Xi _{2},\mho _{2})\in$ q-RLDFHSS($\Re$), then $(\Xi _{1},\mho _{1})^{c}$, $(\Xi _{1},\mho _{1})\cap (\Xi _{2},\mho _{2})$ and $(\Xi _{1},\mho _{1})\cup (\Xi _{2},\mho _{2})$ are also a q-RLDFHSS over $\Re$.

### Proof

Proofs can be easily obtained by the Definitions 0.16,0.15,0.13 respectively $\square$

### Theorem 0.18

Let $(\Xi ,\mho _{1})\in$ q-RLDFHSS($\Re$), then

(i) $$(\Xi ,\mho _{1})_{U}\cup (\Xi ,\mho _{1})$$= $$(\Xi ,\mho _{1})_{U}$$
(ii) $$(\Xi ,\mho _{1})_{\emptyset }\cup (\Xi ,\mho _{1})$$= $$(\Xi ,\mho _{1})$$
(iii) $$(\Xi ,\mho _{1})_{U}\cap (\Xi ,\mho _{1})$$=$$(\Xi ,\mho _{1})$$
(iv) $$(\Xi ,\mho _{1})_{\emptyset }\cap (\Xi ,\mho _{1})$$=$$(\Xi ,\mho _{1})_{\emptyset }$$.

### Proof

proof is obvious $\square$

### Theorem 0.19

Let $(\Xi _{1},\mho _{1}),(\Xi _{2},\mho _{2}),(\Xi _{3},\mho _{3})\in$ q-RLDFHSS($\Re$), then these Associative Law holds

(i) $$((\Xi _{1},\mho _{1})\cup (\Xi _{2},\mho _{2}))\cup (\Xi _{3},\mho _{3})$$= $$(\Xi _{1},\mho _{1})\cup ((\Xi _{2},\mho _{2})\cup (\Xi _{3},\mho _{3}))$$
(ii) $$((\Xi _{1},\mho _{1})\cap (\Xi _{2},\mho _{2}))\cap (\Xi _{3},\mho _{3})$$= $$(\Xi _{1},\mho _{1})\cap ((\Xi _{2},\mho _{2})\cap (\Xi _{3},\mho _{3}))$$.

### Proof

proof is obvious $\square$

### Theorem 0.20

Let $(\Xi _{1},\mho _{1}),(\Xi _{2},\mho _{2})\in$ q-RLDFHSS($\Re$), then these De Morgan’s law holds

(i) $$((\Xi _{1},\mho _{1})\cup (\Xi _{2},\mho _{2}))^{c}$$= $$(\Xi _{1},\mho _{1})^{c}\cap (\Xi _{2},\mho _{2})^{c}$$
(ii) $$((\Xi _{1},\mho _{1})\cap (\Xi _{2},\mho _{2}))^{c}$$= $$(\Xi _{1},\mho _{1})^{c}\cup (\Xi _{2},\mho _{2})^{c}$$.

### Proof

proof is obvious $\square$

### Entropy on q-RLDFHSS

#### Definition 0.21

A real valued map ${\mathscr {E}}: q-RLDFHSS(\Re ) \rightarrow [0,1]$ is said to be ENT on q-RLDFHSS, if ${\mathscr {E}}$ satisfies the conditions

(i) $${\mathscr {E}}(\Xi ,\mho _{1})$$=0 $$\Leftrightarrow (\Xi ,\mho _{1})$$ is HSS
(ii) $${\mathscr {E}}(\Xi ,\mho _{1})$$=1 $$\Leftrightarrow \Delta _{\Xi (\omega _{s})}({\mathfrak {r}}_{p})=\nabla _{\Xi (\omega _{s})}({\mathfrak {r}}_{p})$$ and $$\ltimes _{\Xi (\omega _{s})}({\mathfrak {r}}_{p})=\rtimes _{\Xi (\omega _{s})}({\mathfrak {r}}_{p}),\;\forall \; {\mathfrak {r}}_{p}\in \Re ,\;\omega _{s}\in \mho _{1}$$
(iii) $${\mathscr {E}}(\Xi ,\mho _{1})$$= $${\mathscr {E}}(\Xi ,\mho _{1})^{c}$$
(iv) $${\mathscr {E}}(\Xi _{1},\mho _{1}) \le {\mathscr {E}}(\Xi _{2},\mho _{1})$$ if $$\Delta _{\Xi _{1}(\omega _{s})}({\mathfrak {r}}_{p}) \le \Delta _{\Xi _{2}(\omega _{s})}({\mathfrak {r}}_{p}), \nabla _{\Xi _{2}(\omega _{s})}({\mathfrak {r}}_{p}) \le \nabla _{\Xi _{1}(\omega _{s})}({\mathfrak {r}}_{p})$$,

$$\rtimes _{\Xi _{1}(\omega _{s})}({\mathfrak {r}}_{p}) \le \rtimes _{\Xi _{2}(\omega _{s})}({\mathfrak {r}}_{p})$$ and $$\ltimes _{\Xi _{2}(\omega _{s})}({\mathfrak {r}}_{p}) \le \ltimes _{\Xi _{1}(\omega _{s})}({\mathfrak {r}}_{p})$$

for $$\Delta _{\Xi _{2}(\omega _{s})}({\mathfrak {r}}_{p}) \le \nabla _{\Xi _{2}(\omega _{s})}({\mathfrak {r}}_{p})$$ and $$\rtimes _{\Xi _{2}(\omega _{s})}({\mathfrak {r}}_{p}) \le \ltimes _{\Xi _{2}(\omega _{s})}({\mathfrak {r}}_{p})$$

or $$\Delta _{\Xi _{1}(\omega _{s})}({\mathfrak {r}}_{p}) \ge \Delta _{\Xi _{2}(\omega _{s})}({\mathfrak {r}}_{p}), \nabla _{\Xi _{2}(\omega _{s})}({\mathfrak {r}}_{p}) \ge \nabla _{\Xi _{1}(\omega _{s})}({\mathfrak {r}}_{p})$$,

$$\rtimes _{\Xi _{1}(\omega _{s})}({\mathfrak {r}}_{p}) \ge \rtimes _{\Xi _{2}(\omega _{s})}({\mathfrak {r}}_{p})$$ and $$\ltimes _{\Xi _{2}(\omega _{s})}({\mathfrak {r}}_{p}) \ge \ltimes _{\Xi _{1}(\omega _{s})}({\mathfrak {r}}_{p})$$ for

$$\Delta _{\Xi _{2}(\omega _{s})}({\mathfrak {r}}_{p}) \ge \nabla _{\Xi _{2}(\omega _{s})}({\mathfrak {r}}_{p})$$ and $$\rtimes _{\Xi _{2}(\omega _{s})}({\mathfrak {r}}_{p}) \ge \ltimes _{\Xi _{2}(\omega _{s})}({\mathfrak {r}}_{p})$$

#### Theorem 0.22

Let $\Re =\{{\mathfrak {r}}_{1}, {\mathfrak {r}}_{1},...,{\mathfrak {r}}_{m}\}$ be the universal set and $\mho _{1}=\{\omega _{1}, \omega _{1},..., \omega _{k}\}$ be the set of parameters. Hence $(\Xi ,\mho _{1})=\{\Xi (\omega _{s})$= $\{ \langle \Delta _{\Xi (\omega _{s})}({\mathfrak {r}}_{p}),\nabla _{\Xi (\omega _{s})}({\mathfrak {r}}_{p}) \rangle , \langle \rtimes _{\Xi (\omega _{s})}({\mathfrak {r}}_{p}),\ltimes _{\Xi (\omega _{s})}({\mathfrak {r}}_{p}) \rangle | {\mathfrak {r}}_{p} \in \Re \;and\;\omega _{s} \in \mho _{1}| s=1,2,...,k and p=1,2,...,m \}$ is a family of q-RLDFHSS.

Define ${\mathscr {E}}(\Xi ,\mho _{1})$ as follows:

$${\mathscr {E}}(\Xi ,\mho _{1})$$= $$1-\frac{1}{2mk}\sum \limits _{s=1}^{k} \sum \limits _{p=1}^{m}(|\Delta _{\Xi (\omega _{s})}^{q}({\mathfrak {r}}_{p})-\nabla _{\Xi (\omega _{s})}^{q}({\mathfrak {r}}_{p})| + |\rtimes _{\Xi (\omega _{s})}^{q}({\mathfrak {r}}_{p})-\ltimes _{\Xi (\omega _{s})}^{q}({\mathfrak {r}}_{p})|)$$

#### Proof

We show that ${\mathscr {E}}(\Xi ,\mho _{1})$ satisfies the conditions in Definition 0.21

$$\begin{aligned} (i) {\mathscr {E}}(\Xi ,\mho _{1})=0&\Leftrightarrow 1-\frac{1}{2mk}\sum \limits _{s=1}^{k} \sum \limits _{p=1}^{m}(|\Delta _{\Xi (\omega _{s})}^{q}({\mathfrak {r}}_{p})-\nabla _{\Xi (\omega _{s})}^{q}({\mathfrak {r}}_{p})| + |\rtimes _{\Xi (\omega _{s})}^{q}({\mathfrak {r}}_{p})-\ltimes _{\Xi (\omega _{s})}^{q}({\mathfrak {r}}_{p})|)=0\\&\Leftrightarrow 2-(|\Delta _{\Xi (\omega _{s})}^{q}({\mathfrak {r}}_{p})-\nabla _{\Xi (\omega _{s})}^{q}({\mathfrak {r}}_{p})| + |\rtimes _{\Xi (\omega _{s})}^{q}({\mathfrak {r}}_{p})-\ltimes _{\Xi (\omega _{s})}^{q}({\mathfrak {r}}_{p})|)=0\\&\Leftrightarrow |\Delta _{\Xi (\omega _{s})}^{q}({\mathfrak {r}}_{p})-\nabla _{\Xi (\omega _{s})}^{q}({\mathfrak {r}}_{p})|=1\;and\;|\rtimes _{\Xi (\omega _{s})}^{q}({\mathfrak {r}}_{p})-\ltimes _{\Xi (\omega _{s})}^{q}({\mathfrak {r}}_{p})|=1\\&\Leftrightarrow (\Xi ,\mho _{1}) is HSS \\ (ii) {\mathscr {E}}(\Xi ,\mho _{1})=1&\Leftrightarrow 1-\frac{1}{2mk}\sum \limits _{s=1}^{k} \sum \limits _{p=1}^{m}(|\Delta _{\Xi (\omega _{s})}^{q}({\mathfrak {r}}_{p})-\nabla _{\Xi (\omega _{s})}^{q}({\mathfrak {r}}_{p})| + |\rtimes _{\Xi (\omega _{s})}^{q}({\mathfrak {r}}_{p})-\ltimes _{\Xi (\omega _{s})}^{q}({\mathfrak {r}}_{p})|)=1\\&\Leftrightarrow \sum \limits _{s=1}^{k} \sum \limits _{p=1}^{m}(|\Delta _{\Xi (\omega _{s})}^{q}({\mathfrak {r}}_{p})-\nabla _{\Xi (\omega _{s})}^{q}({\mathfrak {r}}_{p})| + |\rtimes _{\Xi (\omega _{s})}^{q}({\mathfrak {r}}_{p})-\ltimes _{\Xi (\omega _{s})}^{q}({\mathfrak {r}}_{p})|)=0\\&\Leftrightarrow |\Delta _{\Xi (\omega _{s})}^{q}({\mathfrak {r}}_{p})-\nabla _{\Xi (\omega _{s})}^{q}({\mathfrak {r}}_{p})|=0 \;and \;|\rtimes _{\Xi (\omega _{s})}^{q}({\mathfrak {r}}_{p})-\ltimes _{\Xi (\omega _{s})}^{q}({\mathfrak {r}}_{p})|=0\\&\Leftrightarrow \Delta _{\Xi (\omega _{s})}({\mathfrak {r}}_{p})=\nabla _{\Xi (\omega _{s})}({\mathfrak {r}}_{p}) \;and \;\ltimes _{\Xi (\omega _{s})}({\mathfrak {r}}_{p})=\rtimes _{\Xi (\omega _{s})}({\mathfrak {r}}_{p}) \end{aligned}$$

(iii) For $(\Xi ,\mho _{1})\in$ q-RLDFHSS($\Re$) we have

$${\mathscr {E}}(\Xi ,\mho _{1})$$= $$1-\frac{1}{2mk}\sum \limits _{s=1}^{k} \sum \limits _{p=1}^{m}(|\Delta _{\Xi (\omega _{s})}^{q}({\mathfrak {r}}_{p})-\nabla _{\Xi (\omega _{s})}^{q}({\mathfrak {r}}_{p})| + |\rtimes _{\Xi (\omega _{s})}^{q}({\mathfrak {r}}_{p})-\ltimes _{\Xi (\omega _{s})}^{q}({\mathfrak {r}}_{p})|)$$= $${\mathscr {E}}(\Xi ,\mho _{1})^{c}$$

(iv) When $$\Delta _{\Xi _{1}(\omega _{s})}({\mathfrak {r}}_{p}) \le \Delta _{\Xi _{2}(\omega _{s})}({\mathfrak {r}}_{p}), \nabla _{\Xi _{2}(\omega _{s})}({\mathfrak {r}}_{p}) \le \nabla _{\Xi _{1}(\omega _{s})}({\mathfrak {r}}_{p}), \rtimes _{\Xi _{1}(\omega _{s})}({\mathfrak {r}}_{p}) \le \rtimes _{\Xi _{2}(\omega _{s})}({\mathfrak {r}}_{p})$$

and $\ltimes _{\Xi _{2}(\omega _{s})}({\mathfrak {r}}_{p}) \le \ltimes _{\Xi _{1}(\omega _{s})}({\mathfrak {r}}_{p})$ for $\Delta _{\Xi _{2}(\omega _{s})}({\mathfrak {r}}_{p}) \le \nabla _{\Xi _{2}(\omega _{s})}({\mathfrak {r}}_{p})$ and $\rtimes _{\Xi _{2}(\omega _{s})}({\mathfrak {r}}_{p}) \le \ltimes _{\Xi _{2}(\omega _{s})}({\mathfrak {r}}_{p})$,

we have 0 $\le \Delta _{\Xi _{1}(\omega _{s})}({\mathfrak {r}}_{p}) \le \Delta _{\Xi _{2}(\omega _{s})}({\mathfrak {r}}_{p}) \le \nabla _{\Xi _{2}(\omega _{s})}({\mathfrak {r}}_{p}) \le \nabla _{\Xi _{1}(\omega _{s})}({\mathfrak {r}}_{p}) \le$ 1 and

0 $$\le \rtimes _{\Xi _{1}(\omega _{s})}({\mathfrak {r}}_{p}) \le \rtimes _{\Xi _{2}(\omega _{s})}({\mathfrak {r}}_{p}) \le \ltimes _{\Xi _{2}(\omega _{s})}({\mathfrak {r}}_{p}) \le \ltimes _{\Xi _{1}(\omega _{s})}({\mathfrak {r}}_{p}) \le $$ 1, then

$$|\Delta _{\Xi _{2}(\omega _{s})}^{q}({\mathfrak {r}}_{p})-\nabla _{\Xi _{2}(\omega _{s})}^{q}({\mathfrak {r}}_{p})| \le |\Delta _{\Xi _{1}(\omega _{s})}^{q}({\mathfrak {r}}_{p})-\nabla _{\Xi _{1}(\omega _{s})}^{q}({\mathfrak {r}}_{p})|$$ and

$$|\rtimes _{\Xi _{2}(\omega _{s})}^{q}({\mathfrak {r}}_{p})-\ltimes _{\Xi _{2}(\omega _{s})}^{q}({\mathfrak {r}}_{p})| \le |\rtimes _{\Xi _{1}(\omega _{s})}^{q}({\mathfrak {r}}_{p})-\ltimes _{\Xi _{1}(\omega _{s})}^{q}({\mathfrak {r}}_{p})|$$

$$\begin{aligned} \Rightarrow&\frac{1}{2mk}\sum \limits _{s=1}^{k} \sum \limits _{p=1}^{m}(|\Delta _{\Xi _{2}(\omega _{s})}^{q}({\mathfrak {r}}_{p})-\nabla _{\Xi _{2}(\omega _{s})}^{q}({\mathfrak {r}}_{p})| + |\rtimes _{\Xi _{2}(\omega _{s})}^{q}({\mathfrak {r}}_{p})-\ltimes _{\Xi _{2}(\omega _{s})}^{q}({\mathfrak {r}}_{p})|)\\ {}&\;\;\;\;\le \frac{1}{2mk}\sum \limits _{s=1}^{k} \sum \limits _{p=1}^{m}(|\Delta _{\Xi _{1}(\omega _{s})}^{q}({\mathfrak {r}}_{p})-\nabla _{\Xi _{1}(\omega _{s})}^{q}({\mathfrak {r}}_{p})| + |\rtimes _{\Xi _{1}(\omega _{s})}^{q}({\mathfrak {r}}_{p})-\ltimes _{\Xi _{1}(\omega _{s})}^{q}({\mathfrak {r}}_{p})|)\\ \Rightarrow&1-\frac{1}{2mk}\sum \limits _{s=1}^{k} \sum \limits _{p=1}^{m}(|\Delta _{\Xi _{2}(\omega _{s})}^{q}({\mathfrak {r}}_{p})-\nabla _{\Xi _{2}(\omega _{s})}^{q}({\mathfrak {r}}_{p})| + |\rtimes _{\Xi _{2}(\omega _{s})}^{q}({\mathfrak {r}}_{p})-\ltimes _{\Xi _{2}(\omega _{s})}^{q}({\mathfrak {r}}_{p})|)\\ {}&\;\;\;\;\ge 1-\frac{1}{2mk}\sum \limits _{s=1}^{k} \sum \limits _{p=1}^{m}(|\Delta _{\Xi _{1}(\omega _{s})}^{q}({\mathfrak {r}}_{p})-\nabla _{\Xi _{1}(\omega _{s})}^{q}({\mathfrak {r}}_{p})| + |\rtimes _{\Xi _{1}(\omega _{s})}^{q}({\mathfrak {r}}_{p})-\ltimes _{\Xi _{1}(\omega _{s})}^{q}({\mathfrak {r}}_{p})|)\\ \Rightarrow&{\mathscr {E}}(\Xi _{1},\mho _{1}) \le {\mathscr {E}}(\Xi _{2},\mho _{1}) \end{aligned}$$

Similarly, when $$\Delta _{\Xi _{1}(\omega _{s})}({\mathfrak {r}}_{p}) \ge \Delta _{\Xi _{2}(\omega _{s})}({\mathfrak {r}}_{p}), \nabla _{\Xi _{2}(\omega _{s})}({\mathfrak {r}}_{p}) \ge \nabla _{\Xi _{1}(\omega _{s})}({\mathfrak {r}}_{p}), \rtimes _{\Xi _{1}(\omega _{s})}({\mathfrak {r}}_{p}) \ge \rtimes _{\Xi _{2}(\omega _{s})}({\mathfrak {r}}_{p})$$

and $\ltimes _{\Xi _{2}(\omega _{s})}({\mathfrak {r}}_{p}) \ge \ltimes _{\Xi _{1}(\omega _{s})}({\mathfrak {r}}_{p})$ for $\Delta _{\Xi _{2}(\omega _{s})}({\mathfrak {r}}_{p}) \ge \nabla _{\Xi _{2}(\omega _{s})}({\mathfrak {r}}_{p})$ and $\rtimes _{\Xi _{2}(\omega _{s})}({\mathfrak {r}}_{p}) \ge \ltimes _{\Xi _{2}(\omega _{s})}({\mathfrak {r}}_{p})$,

we have $${\mathscr {E}}(\Xi _{1},\mho _{1}) \le {\mathscr {E}}(\Xi _{2},\mho _{1})$$

      Hence proved $$\square $$

## Application

This section presents an MADM Algorithm based on the proposed ENT and an application for selecting suitable WWTT is discussed.

**Algorithm**

Let ${\mathfrak {Y}}=\{{\mathfrak {y}}_{1},{\mathfrak {y}}_{2},...,{\mathfrak {y}}_{w}\}$ be a set of alternatives, suppose $\mho _{1}$= ${\mathfrak {E}}_{1} \times {\mathfrak {E}}_{2} \times ...\times {\mathfrak {E}}_{n}$, where ${\mathfrak {E}}_{1}, {\mathfrak {E}}_{2},..., {\mathfrak {E}}_{n}$ be the corresponding attribute values of n different attributes ${\mathfrak {e}}_{1}, {\mathfrak {e}}_{2},..., {\mathfrak {e}}_{n}$ respectively. The recommended q-RLDFHSS-based ENT was developed in the phases listed below.

Step 1: Each of the q-RLDFHSS should be stated.

Step 2: Determine ENT for each q-RLDFHSS using the formula.

$${\mathscr {E}}(\Xi ,\mho _{1})$$= $$1-\frac{1}{2mk}\sum \limits _{s=1}^{k} \sum \limits _{p=1}^{m}(|\Delta _{\Xi (\omega _{s})}^{q}({\mathfrak {r}}_{p})-\nabla _{\Xi (\omega _{s})}^{q}({\mathfrak {r}}_{p})| + |\rtimes _{\Xi (\omega _{s})}^{q}({\mathfrak {r}}_{p})-\ltimes _{\Xi (\omega _{s})}^{q}({\mathfrak {r}}_{p})|)$$

Step 3: Select a q-RLDFHSS with the lowest ENT and choose it for the best possible outcome.

Step 4: If it received more than one optimum, select any one.

**Figure 2:**
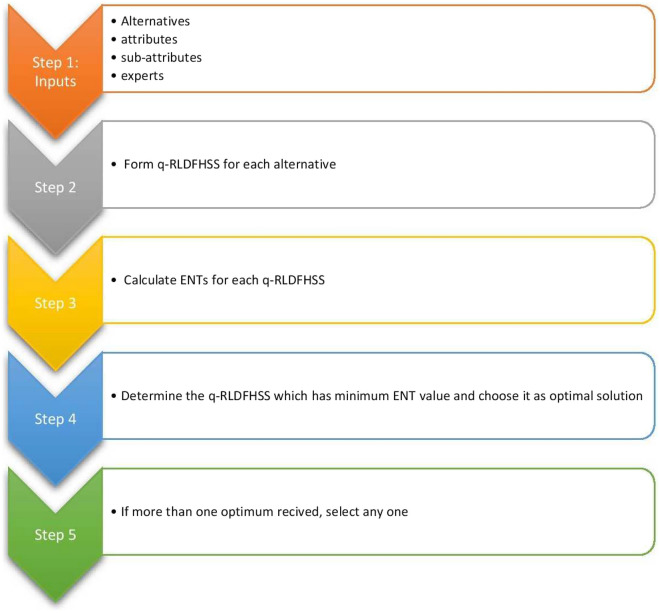
Flowchart of the proposed algorithm.

The algorithm is expressed as a flowchart in Fig. 2.

**Numerical example**

**A general outlook about WWTT**

The removal of impurities from sewage or wasted water to create pollutant that can be recycled back into the water cycle with a minimal negative impact on the environment is known as WWT. It typically has four progressively more difficult levels: (i) preliminary treatment used to treat coarse particles using grits, barracks, or grinders. (ii) Primary treatment used to remove sedimentary sediments and organic matter by gravity. (iii) The removal of coliforms, particulate matter and residual solids. is facilitated by secondary treatment and (iv) nutrients and other micropollutants are eliminated during tertiary treatment. Various WWTT exist, such as Membrane Bioreactor (MBR), Sequential Batch Reactor(SBR) and Fluidized Aerobic Bed Reactor(FAB).

MBR combine biological wastewater treatment methods like activated sludge with membrane processes, such as ultrafiltration or microfiltration. Currently, it is widely employed for the treatment of municipal and industrial wastewater. A submerged MBR (SMBR) and a side stream MBR are the two fundamental MBR variants. In a side stream MBR, after biological treatment, the membrane is situated externally to the reactor as a further stage, but in an SMBR design the membrane is positioned within the biological reactor and submerged in the effluent. The concept of immersing the membranes in the bioreactor by Yamamoto and colleagues led to the MBR’s breakthrough in 1989. Up until that point, MBRs were counted at high trans-membrane pressure (TMP) to keep the filtration going and had a separation mechanism outside the reactor (side stream MBR). Submerged MBR systems, as opposed to sidestream configurations, directly submerge the membrane in the bioreactor.

For wastewater treatment, sequential batch reactors (SBR) or sequencing batch reactors are a form of activated sludge technology. Batch treatment of wastewater from anaerobic digesters, mechanical biological treatment plants, or sewage is carried out in SBR reactors. To decrease the organic content, oxygen was bubbled through the wastewater and activated sludge solution (measured as chemical oxygen demand (COD) and biochemical oxygen demand (BOD)). Cleaned waste can be used on land or dumped into surface waters. SBRs have a variety of configurations, but the fundamental procedure is the same for each. The apparatus comprises of one or more tanks with plug flow or fully mixed reactor capabilities. Raw sewage (influent) enters the tanks through one end, and cleaned water (effluent) exits the other end, forming a “flow through” system. In systems with more than one tank, the other tank is aerating and filling, whereas the first tank is in the settle/decant mode. Some systems have a part of the tank called the bio-selector, which is composed of walls or baffles that direct the flow either under or over successive baffles or from side to side of the tank. Before the spirits enter the tank’s main compartment, the returned activated sludge (RAS) and incoming influent are combined, starting the biological process of digestion.

The most recent development in attached growth aerobic biological treatment technology is the Fluidized Bed Reactor(FBR) procedure. The FBR uses neutrally buoyant bio film carrier elements known as RING PAC MEDIA to produce exceptional BOD/COD removal productivity from a small bioreactor. The liquid that requires treatment is circulated through a bed of tiny media in fluidized bed reactors at a sufficiently fast rate to achieve fluidization. When the media are fluidized, they offer a sizable specific surface for the associated biological growth and permit the development of biomass concentrations between 10 and 40 kg/m3. The reactor is aerated for aerobic treatment procedures. This is accomplished by transferring the liquid through an oxygenator, where air or oxygen may be bubbled, from the reactor. The reactor may be directly aerated to avoid issues with high recirculation rates, which are necessary when there is a high demand for oxygen in the reactor. The immobilization of germs on solid surfaces serves as the foundation for the use of fluidized bed systems. Many bacterial species, as well as other microbes, can adhere to support matrices. A volume of Ring Pac media is submerged in water in this procedure, and is fluidized (kept in motion) by the treatment reactor’s flow of gas and liquid.

Several studies^43–46^ have been conducted to identify acceptable WWTT for various sectors. We recommend selecting a suitable WWTT utilizing the suggested technique to do so more effectively.

**Problem**

Assume an industrial company wants to select a suitable WWTT from the set of WWTT $\{MBR, SBR, FAB\}$ and to analyze these WWTT the industrial company considers the three distinct attributes ${\mathfrak {e}}_{1}$= Economic, ${\mathfrak {e}}_{2}$= socio-political and ${\mathfrak {e}}_{3}$= Environmental whose sub-attributes are ${\mathfrak {E}}_{1}=\{$ capital investment$({\mathfrak {e}}_{11})$, operational cost$({\mathfrak {e}}_{12})\}$, ${\mathfrak {E}}_{2}=\{$ government support$({\mathfrak {e}}_{21})$, social acceptance$({\mathfrak {e}}_{22})\}$ and ${\mathfrak {E}}_{1}=\{$ water quality improvement$({\mathfrak {e}}_{31})\}$ respectively.

Further, for better understanding of the sub-attributes, the description of the sub-attributes is given in Table 3.

**Table 3:** Descriptions of sub-attributes.

Attributes	Sub-attributes	Description
Cost	Capital investment	It indicates the initial investment in all infrastructures to deploy WWTT
	Operational cost	It refers to expenses associated with everyday operations and maintenance, including human resources and energy use for applying WWTT
Socio-political	Government support	It refers to the support from government policies, including economic and regulatory assistance, to facilitate the implementation of certain WWTT.
	Social acceptance	It refers to the support obtained from the people where the WWTT is implemented
Environment	Water quality improvement	It assesses quality improvement and treatment efficiency for removing waste and hazardous materials

Then the cartesian product of sub-attributes is obtained as

$$\mho _{1}$$= $${\mathfrak {E}}_{1} \times {\mathfrak {E}}_{2} \times {\mathfrak {E}}_{3}$$ =$$\{\omega _{1}=({\mathfrak {e}}_{11},{\mathfrak {e}}_{21},{\mathfrak {e}}_{31}), \omega _{2}=({\mathfrak {e}}_{11},{\mathfrak {e}}_{22},{\mathfrak {e}}_{31}), \omega _{3}=({\mathfrak {e}}_{12},{\mathfrak {e}}_{21},{\mathfrak {e}}_{31}), \omega _{4}=({\mathfrak {e}}_{12},{\mathfrak {e}}_{22},{\mathfrak {e}}_{31})\}$$

The classification of attributes is given as follows

- The attribute “Economic” and its attribute values illustrates that the alternative is cheap or not cheap.
- The attribute “socio-political” and its attribute values illustrates that the alternative is maximum or minimum.
- The attribute “Environmental” and its attribute values illustrates that the alternative is good or not good.

Then the cartesian product of sub-attributes illustrates that the alternative is (cheap, maximum, good) all together or (not cheap, minimum, not good) all together

and three decision makers $\{{\mathfrak {y}}_{1},{\mathfrak {y}}_{2},{\mathfrak {y}}_{3}\}$ were set to evaluate the WWTT according to the cartesian product of the sub-attributes, then with the help of these decision makers their opinion and informations are encoded in the form of q-RLDFHSS

The characteristic of these q-RLDFHSSs is $(\langle$ MG, NMG $\rangle , \langle$ (cheap, maximum, good), (not cheap, minimum, not good)$\rangle )$ $\forall \omega _{s}\in \mho _{1}$.

Step 1:

$$\begin{aligned} MBR=(\Xi _{1},\mho _{1})=&\Biggl \{\biggl \langle \omega _{1},\left( \frac{{\mathfrak {y}}_{1}}{\langle (0.88,0.75),(0.85,0.72)\rangle }, \frac{{\mathfrak {y}}_{2}}{\langle (0.73,0.56),(0.54,0.47)\rangle }, \frac{{\mathfrak {y}}_{3}}{\langle (0.82,0.63),(0.61,0.52)\rangle }\right) \biggl \rangle ,\\&\;\;\biggl \langle \omega _{2},\left( \frac{{\mathfrak {y}}_{1}}{\langle (0.83,0.61),(0.72,0.66)\rangle }, \frac{{\mathfrak {y}}_{2}}{\langle (0.74,0.61),(0.73,0.52)\rangle }, \frac{{\mathfrak {y}}_{3}}{\langle (0.79,0.63),(0.48,0.44)\rangle }\right) \biggl \rangle ,\\&\;\;\biggl \langle \omega _{3},\left( \frac{{\mathfrak {y}}_{1}}{\langle (0.71,0.67),(0.62,0.53)\rangle }, \frac{{\mathfrak {y}}_{2}}{\langle (0.59,0.48),(0.6,0.32)\rangle }, \frac{{\mathfrak {y}}_{3}}{\langle (0.68,0.63),(0.53,0.51)\rangle }\right) \biggl \rangle ,\\&\;\;\biggl \langle \omega _{4},\left( \frac{{\mathfrak {y}}_{1}}{\langle (0.67,0.47),(0.66,0.49)\rangle }, \frac{{\mathfrak {y}}_{2}}{\langle (0.57,0.34),(0.6,0.4)\rangle }, \frac{{\mathfrak {y}}_{3}}{\langle (0.5,0.4),(0.4,0.3)\rangle }\right) \biggl \rangle \Biggl \} \\ SBR=(\Xi _{2},\mho _{1})=&\Biggl \{\biggl \langle \omega _{1},\left( \frac{{\mathfrak {y}}_{1}}{\langle (0.82,0.78),(0.78,0.74)\rangle }, \frac{{\mathfrak {y}}_{2}}{\langle (0.65,0.54),(0.48,0.48)\rangle }, \frac{{\mathfrak {y}}_{3}}{\langle (0.75,0.68),(0.58,0.53)\rangle }\right) \biggl \rangle ,\\&\;\;\biggl \langle \omega _{2},\left( \frac{{\mathfrak {y}}_{1}}{\langle (0.77,0.66),(0.70,0.69)\rangle }, \frac{{\mathfrak {y}}_{2}}{\langle (0.7,0.64),(0.7,0.55)\rangle }, \frac{{\mathfrak {y}}_{3}}{\langle (0.75,0.65),(0.43,0.49)\rangle }\right) \biggl \rangle ,\\&\;\;\biggl \langle \omega _{3},\left( \frac{{\mathfrak {y}}_{1}}{\langle (0.67,0.67),(0.59,0.55)\rangle }, \frac{{\mathfrak {y}}_{2}}{\langle (0.54,0.52),(0.56,0.4)\rangle }, \frac{{\mathfrak {y}}_{3}}{\langle (0.64,0.63),(0.51,0.51)\rangle }\right) \biggl \rangle ,\\&\;\;\biggl \langle \omega _{4},\left( \frac{{\mathfrak {y}}_{1}}{\langle (0.61,0.52),(0.6,0.55)\rangle }, \frac{{\mathfrak {y}}_{2}}{\langle (0.52,0.41),(0.44,0.42)\rangle }, \frac{{\mathfrak {y}}_{3}}{\langle (0.46,0.42),(0.36,0.33)\rangle }\right) \biggl \rangle \Biggl \} \\ FAB=(\Xi _{3},\mho _{1})=&\Biggl \{\biggl \langle \omega _{1},\left( \frac{{\mathfrak {y}}_{1}}{\langle (0.8,0.78),(0.76,0.75)\rangle }, \frac{{\mathfrak {y}}_{2}}{\langle (0.61,0.57),(0.47,0.51)\rangle }, \frac{{\mathfrak {y}}_{3}}{\langle (0.7,0.69),(0.56,0.54)\rangle }\right) \biggl \rangle ,\\&\;\;\biggl \langle \omega _{2},\left( \frac{{\mathfrak {y}}_{1}}{\langle (0.73,0.69),(0.68,0.71)\rangle }, \frac{{\mathfrak {y}}_{2}}{\langle (0.67,0.65),(0.62,0.56)\rangle }, \frac{{\mathfrak {y}}_{3}}{\langle (0.71,0.68),(0.45,0.47)\rangle }\right) \biggl \rangle ,\\&\;\;\biggl \langle \omega _{3},\left( \frac{{\mathfrak {y}}_{1}}{\langle (0.67,0.7),(0.55,0.55)\rangle }, \frac{{\mathfrak {y}}_{2}}{\langle (0.53,0.53),(0.49,0.41)\rangle }, \frac{{\mathfrak {y}}_{3}}{\langle (0.63,0.63),(0.48,0.53)\rangle }\right) \biggl \rangle ,\\&\;\;\biggl \langle \omega _{4},\left( \frac{{\mathfrak {y}}_{1}}{\langle (0.58,0.53),(0.57,0.56)\rangle }, \frac{{\mathfrak {y}}_{2}}{\langle (0.49,0.43),(0.44,0.42)\rangle }, \frac{{\mathfrak {y}}_{3}}{\langle (0.44,0.42),(0.35,0.33)\rangle }\right) \biggl \rangle \Biggl \} \end{aligned}$$

We will assume that q=3

In the q-RLDFHSS of WWTT MBR,decision maker ${\mathfrak {y}}_{1}$ and parameter $\omega _{1}$=(capital investment, government support, water quality improvement) have the numeric value $\langle (0.88,0.75), (0.85,0.72)\rangle$. This value indicates that for MBR the decision maker ${\mathfrak {y}}_{1}$ articulates 88% of membership and 75% of non membership under the parameter $\omega _{1}$. The pair (0.85,0.72) represents the RPs of membership and non membership respectively, where we can study that for (cheap at capital investment, maximum at government support, good at water quality improvement) all together the decision maker ${\mathfrak {y}}_{1}$ articulates 85% to MBR and for (not cheap at capital investment, minimum at government support, not good at water quality improvement) all together the decision maker ${\mathfrak {y}}_{1}$ articulates 72% to MBR. In a similar fashion all the values in the q-RLDFHSSs MBR, SBR and FAB are articulated.

Step 2: Calculate the ENTs of $(\Xi _{1},\mho _{1})$, $(\Xi _{2},\mho _{1})$ and $(\Xi _{3},\mho _{1})$ using the proposed ENT of q-RLDFHSS, Hence the entropies are ${\mathscr {E}}(\Xi _{1},\mho _{1})$= 0.852136458, ${\mathscr {E}}(\Xi _{2},\mho _{1})$= 0.939666083 and ${\mathscr {E}}(\Xi _{3},\mho _{1})$= 0.972535166 respectively.

Step 3: The best choice is $(\Xi _{1},\mho _{1})$ as it has the lowest ENT value.

From the result we obtain that MBR is the suitable WWTT for the industry followed by SBR and then FAB.

## Comparative study

**Validity test**

The link between alternatives, the consistency of the attributes, and the objective assessments of the decision-makers all affect how a MADM technique works. For a MADM technique to be deemed valid, Wang and Triantaphyllou^47^ developed three efficient validity test criteria. The following are the test criteria.

**Test criteria 1**: When a non-optimal alternative is substituted with a worse alternative without affecting the significance of each attribute, the best alternative remains the same.

**Test criteria 2**: A useful MADM technique should have the transitive nature.

**Test criteria 3**: When we split the MADM problem into smaller sub-problems, the order of the smaller sub-problems must match the order of the original problem.

The proposed method’s validity is investigated as follows:

Assume three alternatives ${\mathfrak {U}}_{1}, {\mathfrak {U}}_{2}, {\mathfrak {U}}_{3}$ by considering the same attributes and information taken in problem 4.2.2 then the respective q-RLDFHSSs of alternatives ${\mathfrak {U}}_{1}, {\mathfrak {U}}_{2}, {\mathfrak {U}}_{3}$ will be also the same as in the problem 4.2.2, i.e) q-RLDFHSS of ${\mathfrak {U}}_{1}$ is $(\Xi _{1},\mho _{1})$, ${\mathfrak {U}}_{2}$ is $(\Xi _{2},\mho _{1})$ and ${\mathfrak {U}}_{3}$ is $(\Xi _{3},\mho _{1})$. Now using the proposed method the alternatives got ranked as ${\mathfrak {U}}_{1}> {\mathfrak {U}}_{2}>{\mathfrak {U}}_{3}$.

Now lets analyze the test criteria for this problem.

**Test criteria 1**: Consider the non-optimal alternative ${\mathfrak {U}}_{3}$ and substitute with a worse alternative $\widehat{{\mathfrak {U}}_{3}}$. Suppose the q-RLDFHSS of the alternative $\widehat{{\mathfrak {U}}_{3}}$ is

$$\begin{aligned} \widehat{{\mathfrak {U}}_{3}}=(\widehat{\Xi _{3}},\mho _{1})=&\Biggl \{\biggl \langle \omega _{1},\left( \frac{{\mathfrak {y}}_{1}}{\langle (0.77,0.8),(0.75,0.77)\rangle }, \frac{{\mathfrak {y}}_{2}}{\langle (0.58,0.60),(0.48,0.51)\rangle }, \frac{{\mathfrak {y}}_{3}}{\langle (0.68,0.71),(0.53,0.55)\rangle }\right) \biggl \rangle ,\\&\;\;\biggl \langle \omega _{2},\left( \frac{{\mathfrak {y}}_{1}}{\langle (0.68,0.7),(0.7,0.71)\rangle }, \frac{{\mathfrak {y}}_{2}}{\langle (0.64,0.65),(0.59,0.59)\rangle }, \frac{{\mathfrak {y}}_{3}}{\langle (0.69,0.7),(0.44,0.47)\rangle }\right) \biggl \rangle ,\\&\;\;\biggl \langle \omega _{3},\left( \frac{{\mathfrak {y}}_{1}}{\langle (0.67,0.7),(0.53,0.55)\rangle }, \frac{{\mathfrak {y}}_{2}}{\langle (0.51,0.54),(0.46,0.44)\rangle }, \frac{{\mathfrak {y}}_{3}}{\langle (0.6,0.61),(0.49,0.52)\rangle }\right) \biggl \rangle ,\\&\;\;\biggl \langle \omega _{4},\left( \frac{{\mathfrak {y}}_{1}}{\langle (0.54,0.55),(0.55,0.58)\rangle }, \frac{{\mathfrak {y}}_{2}}{\langle (0.47,0.45),(0.42,0.44)\rangle }, \frac{{\mathfrak {y}}_{3}}{\langle (0.41,0.44),(0.3,0.33)\rangle }\right) \biggl \rangle \Biggl \} \end{aligned}$$

When the proposed method is applied, we obtain the new alternative ranking as ${\mathfrak {U}}_{1}> {\mathfrak {U}}_{2}>\widehat{{\mathfrak {U}}_{3}}$. It seems that the optimal alternative has remained the same. As a result, test criteria 1 is valid.

**Test criteria 2 and 3**: We split the MADM problem into sub-problems: $\{{\mathfrak {U}}_{1}, {\mathfrak {U}}_{2}\}$, $\{{\mathfrak {U}}_{2}, {\mathfrak {U}}_{3}\}$ and $\{{\mathfrak {U}}_{1}, {\mathfrak {U}}_{3}\}$. The rank results of each sub-problem are obtain as ${\mathfrak {U}}_{1}> {\mathfrak {U}}_{2}$, ${\mathfrak {U}}_{2}> {\mathfrak {U}}_{3}$ and ${\mathfrak {U}}_{1}> {\mathfrak {U}}_{3}$ respectively. Thus, the overall ranking can be seen ${\mathfrak {U}}_{1}>{\mathfrak {U}}_{2}> {\mathfrak {U}}_{3}$. Transivity property is thus offered between these rankings. As a result, test criteria 2 and 3 are also valid.

**Comparative analysis**

The comparison of the suggested notion with the other current theories is displayed in Table 4.

**Table 4:** Comparision table.

Notion	MG	NMG	RPs	Attributes	Multi sub-attributes
FS^1^	$\checkmark$	$\times$	$\times$	$\times$	$\times$
IFS^2^	$\checkmark$	$\checkmark$	$\times$	$\times$	$\times$
q-ROFS^4^	$\checkmark$	$\checkmark$	$\times$	$\times$	$\times$
LDFS^5^	$\checkmark$	$\checkmark$	$\checkmark$	$\times$	$\times$
q-RLDFS^8^	$\checkmark$	$\checkmark$	$\checkmark$	$\times$	$\times$
SS^9^	$\times$	$\times$	$\times$	$\checkmark$	$\times$
FSS^10^	$\checkmark$	$\times$	$\times$	$\checkmark$	$\times$
IFSS^11^	$\checkmark$	$\checkmark$	$\times$	$\checkmark$	$\times$
q-ROFSS^13^	$\checkmark$	$\checkmark$	$\times$	$\checkmark$	$\times$
LDFSS^14^	$\checkmark$	$\checkmark$	$\checkmark$	$\checkmark$	$\times$
HSS^15^	$\times$	$\times$	$\times$	$\checkmark$	$\checkmark$
FHSS^15^	$\checkmark$	$\times$	$\times$	$\checkmark$	$\checkmark$
IFHSS^15^	$\checkmark$	$\checkmark$	$\times$	$\checkmark$	$\checkmark$
q-ROFHSS^17^	$\checkmark$	$\checkmark$	$\times$	$\checkmark$	$\checkmark$
q-RLDFHSS(proposed)	$\checkmark$	$\checkmark$	$\checkmark$	$\checkmark$	$\checkmark$

From the comparison table, it can be observed that the proposed q-RLDFHSS outranked the other notions mentioned in the Table 4. Further the superiority of proposed notion over the existing notion is discussed below.

- Although q-RLDFS^8^ is capable of handling issues that are unable to be handled by FS^1^, IFS^2^, PFS^3^, q-ROFS^4^, and LDFS^5^, it is challenging to address q-RLDF issues when they fall under many sub-attributes. The suggested q-RLDFHSS is sufficient for handling q-RLDF issues with many sub-attributes. Furthermore, as current attributed fuzzy notions such as FSS^10^, IFSS^11^, PFSS^12^, q-ROFSS^13^, LDFSS^14^, FHSS^15^, IFHSS^15^, PFHSS^16^, and q-ROFHSS^17^ are unable to handle issues in q-RLDF environments, the suggested q-RLDFHSS is superior to them.

Now to show the superiority of the proposed ENT based MADM algorithm over the existing fuzzy ENT based algorithms the advantages and drawbacks of the existing fuzzy ENT based algorithms are described in Table 5 followed that the proposed ENT based MADM algorithm’s advantages and superiority are discussed.

**Table 5:** Advantages and drawbacks of existing ENT based DM techniques.

ENT	Advantages	Drawbacks
FSS^32^	Has the ability to handle DM Scenarios with FSS by means of measuring fuzziness of FSS	When the handling DM scenario is not restricted by the FS restrictions it is unable to measure the fuzziness with this FSS ENT which makes this DM technique not capable to handle such DM situations. Also, even if the handling real-life DM situation is under FS restriction but need to handle multiple parameters together then this FSS ENT is unable to measure fuzziness in such DM situations
IFS^33^	Has the ability to handle DM Scenarios with IFS by means of measuring fuzziness of IFS	When the handling DM scenario is not restricted by the IFS restrictions it is unable to measure the fuzziness with this IFS ENT which makes this DM technique not capable to handle such DM situations. Also, even if the handling real-life DM situation is under IFS restriction but need to handle real-life parametric situations, this IFS ENT is unable to measure fuzziness in such DM situations
IFSS^34^	Has the ability to handle DM Scenarios with IFSS by means of measuring fuzziness of IFSS	When the handling DM scenario is not restricted by the IFS restrictions it is unable to measure the fuzziness with this FSS ENT which makes this DM technique not capable to handle such DM situations. Also, even if the handling real-life DM situation is under IFS restriction but need to handle multiple parameters together then this IFSS ENT is unable to measure fuzziness in such DM situations
PFS^35^	Has the ability to handle DM Scenarios with PFS by means of measuring fuzziness of PFS	When the handling DM scenario is not restricted by the PFS restrictions it is unable to measure the fuzziness with this PFS ENT which makes this DM technique not capable to handle such DM situations. Also, even if the handling real-life DM situation is under PFS restriction but need to handle real-life parametric situations then this PFS ENT is unable to measure fuzziness in such DM situations
PFSS^36^	Has the ability to handle DM Scenarios with PFSS by means of measuring fuzziness of PFSS	When the handling DM scenario is not restricted by the PFS restrictions it is unable to measure the fuzziness with this PFSS ENT which makes this DM technique not capable to handle such DM situations. Also, even if the handling real-life DM situation is under PFS restriction but need to handle multiple parameters together then this PFSS ENT is unable to measure fuzziness in such DM situations
q-ROFS^37^	Has the ability to handle DM Scenarios with q-ROFS by means of measuring fuzziness of q-ROFS	When the handling DM scenario is not restricted by the q-ROFS restrictions it is unable to measure the fuzziness with this q-ROFS ENT which makes this DM technique not capable to handle such DM situations. Also, even if the handling real-life DM situation is under q-ROFS restriction but need to handle real-life parametric situations then this q-ROFS ENT is unable to measure fuzziness in such DM situations
LDFS^38^	Has the ability to handle DM scenarios with LDFS by means of measuring fuzziness of LDFS	When the handling DM scenarios is not restricted by the LDFS restrictions it is unable to measure the fuzziness with this LDFS ENT which makes this DM technique not capable to handle such DM situations. Also, even if the handling real-life DM situation is under LDFS restriction but need to handle real-life parametric situations then this FSS ENT is unable to measure fuzziness in such DM situations

- From Table 5 we can observe the advantages and drawbacks of existing ENT based DM techniques since the proposed ENT measure can measure fuzziness even when the handling situation is in a multi-sub attributed q-RLDF environment this makes the proposed ENT based DM technique to overcome the drawbacks of existing techniques mentioned in 5, further the other ENT measures^32–39^ that are based on various fuzzy theories are not involved in measuring the fuzziness of problems under these conditions.

Through this research and comparative analysis, we can conclude that the outcomes of the proposed MADM approaches are more robust and universal than those of prevailing procedures. However, the proposed MADM process incorporates more information than current MADM techniques to address the degree of uncertainty of the data. Consequently, compared with previous MADM approaches for various fuzzy structures, our proposed MADM approach is more efficient, adaptable, straightforward, and superior.

**Limitations**

Although the proposed q-RLDFHSS is superior to many current theories, it still has some minimal drawback, such as

- The proposed theory is incapable of handling the case when the tackling situation is not constrained by the q-RLDFS restriction.

## Conclusion and future studies

q-RLDFS is a fresh addition to FS theory for handling uncertain issues across a broad spectrum. In this study, by combining q-RLDFS and HSS together, a novel fuzzy extension named q-RLDFHSS was established, which provides greater promise for solving real-world problems than present fuzzy extensions. Further, several fundamental algebraic operations of q-RLDFHSS such as union, intersection and complement, were also established. Then, the ENT of the proposed q-RLDFHSS was also established to measure the fuzziness of q-RLDFHSSs. To deal with real-life MADM situations more efficiently, this study introduces a MADM algorithm based on the suggested ENT, which has the ability to address real-life MADM situations that are not covered by existing fuzzy theories. The real-world issue of choosing an appropriate WWTT was discussed in relation to the practical application of the proposed algorithm. Also, a comprehensive comparative study has been undertaken in this study, which provides the validity of the proposed algorithm by testing the validity criteria developed by Wang and Triantaphyllou. Following the validity test, a comparative analysis has been provided that compares the proposed notion with the existing fuzzy extensions and also compares the proposed ENT based MADM algorithm with the existing fuzzy ENT based MADM algorithms. Overall, this study highly contributes to the scientific community in both theoretical and practical aspects. whereas when it comes to theory, we can observe that the proposed q-RLDFHSS prevails over the existing fuzzy extensions, and when it comes to solving real-life MADM problems, the study proposes a valid MADM algorithm that has the capacity to tackle real-life MADM scenarios that are not addressed by existing fuzzy theories. But even though the proposed theories and MADM algorithm have lots of advantages, they also exhibit some small drawbacks, such as the suggested theory cannot handle situations that fails to follow the q-RLDFS limitation. So, to overcome the drawbacks of the proposed work as a future work HSS is going to be fused with various other complex and hybrid fuzzy extensions, as stated below as future studies.

**Future studies**

In future studies, to tackle multi sub attributed problems well under different complex and hybrid fuzzy domains, we will merge the HSS theory with various complex and hybrid fuzzy domains such as bipolar complex fuzzy set^48^, fuzzy superior mandelbrot set^49^, complex spherical fuzzy set^50^ and complex T-spherical fuzzy set^51^. In addition, we will work on various DM approaches such as CRITIC^52^, WASPAS^53^ and LOPCOW^54^ to develop and discuss these DM approaches under the proposed notion.

## Data Availability

All data generated or analysed during this study are included in this published article.
